# Post COVID-19 condition is associated with altered regional cerebral blood volume as revealed by dynamic susceptibility contrast MRI

**DOI:** 10.3389/fnimg.2025.1688973

**Published:** 2026-02-18

**Authors:** Bradley J. MacIntosh, S. Shirley Lin, Finn O’Hara, Nathan W. Churchill, Fred Tam, Alexandra Pavel, Eugenie Roudaia, Allison B. Sekuler, Ivy Cheng, Fuqiang Gao, Benjamin Lam, Chris Heyn, Mario Masellis, J. Jean Chen, Tom A. Schweizer, Sandra E. Black, Simon J. Graham

**Affiliations:** 1 Physical Sciences Platform, Sunnybrook Research Institute, Toronto, ON, Canada; 2 Department of Medical Biophysics, University of Toronto, Toronto, ON, Canada; 3 Hurvitz Brain Sciences Program, Sunnybrook Research Institute, Toronto, ON, Canada; 4 Computational Radiology and Artificial Intelligence Unit, Division of Radiology and Nuclear Medicine, Oslo University Hospital, Oslo, Norway; 5 Department of Biomedical Engineering, University of Waterloo, Waterloo, ON, Canada; 6 Neuroscience Research Program, St. Michael’s Hospital, Toronto, ON, Canada; 7 Keenan Research Centre for Biomedical Science, St. Michael’s Hospital, Toronto, ON, Canada; 8 Department of Physics, Toronto Metropolitan University, Toronto, ON, Canada; 9 Rotman Research Insititute, Baycrest Academy for Research and Education, Toronto, ON, Canada; 10 Department of Psychology, University of Toronto, Toronto, ON, Canada; 11 Department of Psychology, Neuroscience and Behaviour, McMaster University, Hamilton, ON, Canada; 12 Evaluative Clinical Sciences, Sunnybrook Research Institute, Toronto, ON, Canada; 13 Integrated Community Program, Sunnybrook Research Institute, Toronto, ON, Canada; 14 Division of Emergency Medicine, Department of Medicine, University of Toronto, Toronto, ON, Canada; 15 LC Campbell Cognitive Neurology Research Group, Sunnybrook Health Sciences Centre, Toronto, ON, Canada; 16 Division of Neurology, Department of Medicine, Sunnybrook Health Sciences Centre, University of Toronto, Toronto, ON, Canada; 17 Department of Medical Imaging, University of Toronto, Toronto, ON, Canada; 18 Department of Biomedical Engineering, University of Toronto, Toronto, ON, Canada; 19 Faculty of Medicine, Division of Neurosurgery, University of Toronto, Toronto, ON, Canada

**Keywords:** cerebral blood volume, post-COVID-19 condition, cerebral blood flow, dynamic susceptibility contrast, magnetic resonance imaging

## Abstract

**Background:**

Coronavirus disease 2019 (COVID-19) has been associated with central nervous system dysfunction implicating cerebrovascular and neurovascular units, as reflected in lower regional cerebral blood flow among non-hospitalized individuals that experienced post COVID-19 condition (PCC). This study investigates whether PCC is associated with altered regional cerebral blood volume assessed using Dynamic Susceptibility Contrast (DSC) Magnetic Resonance Imaging (MRI). The comparison control group are individuals without PCC who previously experienced cold or flu-like symptoms, or COVID-19.

**Methods:**

Fifty-seven participants were recruited: 36 with PCC (mean age: 42.7, standard deviation: 10.4, 26 females) and 21 controls (mean age: 41.6, standard deviation: 14.7, 13 females). T2*-weighted DSC MRI was performed at 3 Tesla to image the first passage of the bolus. A total of 22 regions of interest (ROIs) were considered. Group differences in DSC-derived cerebral blood volume (rCBV) and cerebral blood flow (rCBF) were evaluated using Bayesian regression, providing median group differences, highest density interval (HDI), and the probability of direction (PD) estimates.

**Results:**

The two groups (PCC and controls) were matched for age, sex, days from symptom onset, and number of previous vaccines, but had different degrees of self-report illness severity. The rCBV analysis showed median group differences (range: −0.05 to −0.13), with PD > 0.90, indicating a high probability of decreased rCBV in the PCC group, involving the superior frontal gyrus, thalamus, paracentral lobule, cingulate gyrus, postcentral gyrus, middle frontal gyrus, inferior frontal gyrus, and superior temporal gyrus ROIs. By comparison, group differences in rCBF were muted and did not reach PD > 0.90.

**Discussion:**

We found group-level differences that were reflected by lower regional rCBV in PCC relative to controls. The imaging findings are suggestive of cerebrovascular alterations several months after the initial illness.

## Introduction

Post-COVID-19 condition (PCC) affects 12,700 per 100,000 infected by COVID-19 (Ballering et al., 2022). Across 21 countries, the rate of excess mortality due to the COVID-19 pandemic is 300 deaths per 100,000 (COVID-19 Excess Mortality Collaborators, 2022). PCC refers to the persistence of symptoms at least three months after COVID-19 infection, lasting a minimum of two months (Soriano et al., 2022). Symptoms emerge after initial recovery, persist from the acute illness, or relapse over time (Soriano et al., 2022; Hanson et al., 2022). PCC occurs in a significant portion of individuals who did not require hospitalization (10–30%) or were vaccinated (10–12%) (Davis et al., 2023).

PCC presents with diverse neurological symptoms, including fatigue, headache, attention disorder, dyspnea, ageusia, anosmia, memory loss, and hearing loss/tinnitus, and can significantly impair daily functioning (Soriano et al., 2022; Hanson et al., 2022; Lopez-Leon et al., 2021). The immunological, virological, and neurological implications of PCC point to a potentially widespread diffuse impact on the neurovascular-glial unit (Davis et al., 2023; van der Knaap et al., 2025). Our understanding of the neurophysiological and functional brain metabolism alterations associated with PCC can be informed by advanced neuroimaging techniques (Taquet et al., 2022; Xu et al., 2022).

This NeuroCOVID-19 study was initiated early on during the pandemic and included a unique DSC MRI sequence to investigate neurovascular physiology (Araújo et al., 2023). DSC enables the measurement of relative CBV (rCBV) and rCBF based on the first passage of intravenous bolus administration of Gadolinium-based contrast agent. The objective of the current study was to examine rCBV and rCBF in brain ROIs in a cohort characterized by neuropsychological and self-report measures (MacIntosh et al., 2021). A recent functional and anatomical neuroimaging meta-analysis highlights the importance of considering different imaging approaches to further our understanding of the cerebrovascular associations with PCC (Guo et al., 2024). Prior arterial spin labeling (ASL) MRI work helped to reveal CBF alterations among individuals with COVID-19 (Yus et al., 2022; Qin et al., 2021) and relative to controls (Kim et al., 2023; Ajčević et al., 2023). The current study tests the hypothesis that individuals with PCC exhibit reduced regional rCBV compared to controls. A secondary objective involved equivalent group comparisons based on the DSC-derived rCBF.

## Materials and methods

### Participants

Participants were recruited between May 2020 and December 2023 through community advertisement, physician referral, and the Department of Emergency Medicine at our institute. For context, this hospital is a tertiary-level academic centre with an emergency department that receives 60,000 patients per year. Eligibility and consenting procedures were performed over phone or email. Participants provided written informed consent. The study was approved by the Sunnybrook Health Sciences Centre Research Ethics Board and in accordance with the Declaration of Helsinki.

Participants were 19–70 years of age, had a healthy glomerular filtration rate, were living independently, and had documented evidence from public health or workplace documentation, emails, pictures, text messages, or social media posts to infer the COVID-19 diagnosis. Exclusion criteria included an existing neurological disorder, previous brain injury, severe psychiatric illness, on-going unstable cardiovascular disease, or contraindications to MRI.

### Study design

As described in the NeuroCOVID-19 study protocol (MacIntosh et al., 2021), PCC participants had a prior COVID-19 infection that did not require hospitalization, but subsequently experienced fatigue, shortness of breath, and/or cognitive disturbances over 3 months after they were no longer infectious, with these symptoms lasting at least 2 months with no alternative explanation. Due to the high prevalence of COVID-19 infection, identifying a COVID-19 naïve control group was not feasible. Controls (denoted as C) did not have PCC at the time of brain MRI, but experienced cold and/or flu symptoms, or confirmed or suspected COVID-19 in the past. The current analysis consisted of DSC, pre- and post-contrast T1-weighted anatomical images, symptom self-report, and Cognition and Emotion Battery scores from established National Institutes of Health (NIH) toolboxes, respectively (Gershon et al., 2013; Hodes et al., 2013).

### MRI acquisition

MRI was performed using a 3 T MRI system that was equipped with a 12-channel head coil (Magnetom Prisma, Siemens Healthineers, Erlangen, Germany). T1-weighted MRI was performed before and after the exogeneous contrast agent administration, using a 3D-MPRAGE sequence (1 mm isotropic voxel resolution, repetition time/echo time/inversion time/flip angle (TR/TE/TI/FA) = 2,500 ms/ 4.37 ms/ 1,100 ms/ 7 degrees; 3 min: 45 s acquisition time). The T1-weighted image was repeated ten minutes after DSC. Images were inspected by a research neuroradiologist for incidental findings (F. G.). DSC used single-shot T2*-weighted EPI (220 × 220 mm in-plane field of view, 20 slices, 30% slice gap, voxel resolution of 1.7 × 1.7 × 4.0 mm, TR/TE/FA = 1,250 ms/ 30 ms/ 90 degrees, GeneRalized Autocalibrating Partially Parallel Acquisitions (GRAPPA) acceleration = 3, 140 time points, 3 min: 7 s acquisition time). EPI started prior to the intravenous injection of Gadovist® contrast agent (Bayer HealthCare Pharmaceuticals, Berlin, Germany) using a power injector (Spectris Solaris EP, Medrad Inc., Warren, Pennsylvania, USA). The injection started at the 30th EPI time point (i.e., 37.5 s from the start of acquisition), with an injection rate of 5 cc/s, bolus concentration of 1.0 mmoL/mL, bolus volume of 1 mL/kg (patient weight), and a saline flush volume of 25 mL at 5 cc/s. DSC data were collected without a pre-bolus injection.

### Additional assessments

Participants completed a self-report questionnaire evaluating flu-like symptoms, including fever, cough, sore throat, shortness of breath, headache, fatigue, gastrointestinal symptoms, and changes in smell or taste (see supplementary file). Symptoms were rated as absent, resolved, or currently ongoing. Study staff ensured that reported symptoms in the PCC group occurred after participants initially began to feel unwell, specifically originating from COVID-19.

The NIH Toolbox Cognition Battery (Version 1.23.4940) was administered via a computer tablet (iPad, OS Version 16.4.1), generating age-corrected standardized T-scores (mean = 100, standard deviation = 15) indexing fluid and crystallized cognition. Higher T-scores indicate better cognitive performance relative to age norms. The NIH Toolbox Emotion Battery produced three T-scores (mean = 50, standard deviation = 10) reflecting negative affect, social satisfaction, and well-being. For these emotional measures, higher T-scores correspond to increased intensity of the trait of interest (Hodes et al., 2013; Cella et al., 2010).

### DSC processing

Image preprocessing was performed by staff blinded to the participant group. DSC data were fit to an established tracer kinetic model (Ostergaard et al., 1996). Steps were conducted using analysis of functional neuroimages (AFNI) software (Cox, 1996), including skull-stripping with *3dAutomask*, slice-time correction with *3dTshift*, motion correction with *3dvolreg*, physiological noise correction by bandpass filtering, and semi-automated selection of the arterial input function (AIF, described below). Images were transformed to a standard reference coordinate system to generate voxel-wise time series of contrast agent concentration. DSC model parameters were estimated using block-circulant singular value decomposition, as this approach is reported to be less sensitive to AIF delays and dispersion (Wu et al., 2003; Peruzzo et al., 2017). Open-source (https://github.com/marcocastellaro/dsc-mri-toolbox) and Matlab software (the Mathworks, Inc., Natick, USA) were used for the DSC calculations. The regional rCBV and rCBF estimates were recorded (time to peak and mean transit time were not considered further). Additionally, initial modeling included evaluation of the contrast leakage term. It was observed that modeling contrast extravasation could introduce potential bias on other parameters. Unlike in the cases of acute stroke or brain tumors, it was thus assumed that individuals in the PCC group (none of whom were hospitalized during acute infection) and control groups would not exhibit high vascular permeability of the contrast agent. For these reasons, the leakage term was not included in the final DSC analysis.

AIF selection involved a semi-automated procedure to allow greater control over the anatomical location of the AIF voxels. The angiographic image contrast (i.e., high signal intensity) on post-contrast T1-weighted images was used to locate artery locations on lower slices in the DSC data. This was performed by visual inspection after co-registration of the T1-weighted and DSC images, recognizing that a precise registration would be a challenge due to geometric distortion and thus selecting artery voxels within a bounding box. Candidate AIF voxels on the DSC data were selected independently by one of two experienced users (S. L. L. and F. O.) using the left and right middle cerebral arteries as anatomical landmarks of interest. An AFNI tool was used to visualize voxel time series in a 7 × 7 voxel grid. Six voxels were selected (3 per hemisphere) and averaged to create one AIF per participant.

Analysis was performed for specific brain regions selected from 246 ROIs in the Brainnetome atlas (Yu et al., 2011). Considering the spatial resolution of DSC MRI, regions were merged resulting in 44 coarse ROIs, followed by averaging across hemispheres to yield a final total of 22 ROIs. The rCBV and rCBF estimates were normalized by the primary visual cortex, in line with other work (Yoshiura et al., 2009; Kim et al., 2023).

### Statistical analysis

Demographic and clinical characteristics were compared between groups using unpaired samples t-tests or Mann–Whitney U-tests for continuous data. Chi-squared tests were run on categorical data (e.g., proportion female) and on the histograms of other categorical variables (i.e., symptoms on-going, resolved, or not experienced). The threshold for statistical significance of demographic and clinical variables was set at *p* = 0.05, uncorrected.

The rCBV data were winsorized by limiting values at the 5th and 95th percentiles of each group to reduce the influence of extreme outliers. Bayesian statistics were used, which combined prior beliefs with observed data for probabilistic inferences. The Bayesian prior distribution reflected the range of plausible values before observing the rCBV and producing a group difference probability and confidence intervals.

Bayesian multilevel regression investigated group differences in ROI-based rCBV. This was implemented in the Stan platform for modeling and computation (https://mc-stan.org) with the Bayesian regression package (https://cran.r-project.org/web/packages/brms/index.html) (Bürkner, 2020). Priors were selected via prior predictive simulation checks to examine a credible range of outcomes, and regularize posterior estimates. Posterior distribution estimates were derived using Hamiltonian Monte Carlo simulation in RStan (https://mc-stan.org/docs/2_19/reference-manual/hamiltonian-monte-carlo.html) (Version 2.21). The CalvinBayes (https://github.com/CalvinData/CalvinBayes) package was used for posterior samples and density plots. The rCBV were regressed onto binary predictors of group status (C = 0, PCC = 1) along with the main effects of sex and age. Priors on the model coefficients, intercept, and standard error were selected to be weakly regularizing with a Gaussian distribution (mean = 0, SD = 1) to constrain the estimates to be minimally informative on the observed data. The regression model output provided a joint posterior distribution over the model parameters. Posterior distributions were summarized via the median, and 90% highest density interval (90% HDI), with the latter being the most compact interval containing 90% of the posterior probability mass. These values provided a point and interval estimate for the group difference. Probability of Direction (PD) values were also reported as the fraction of the posterior probability distribution associated with the most probable direction of effect (positive or negative), indicating confidence in the sign of the reported effect. The threshold for significant PD was chosen to be >90%.

Analogous statistical procedures were also conducted for rCBF.

## Results

### Demographic and clinical characteristics

A total of 57 participants (21C, 36 PCC) were involved in the analysis. Table 1 shows the demographic and clinical characteristics, expressed as mean ± standard deviation, median value, or counts (percentage). The groups showed no significant differences in age (C: 41.6 ± 14.7 years; PCC: 42.7 ± 10.4 years), percentage female (C: 62%; PCC: 72%), percentage Caucasian (C: 81%; PCC: 67%), education (C: 17.1 ± 2.8 years; PCC: 16.9 ± 2.7 years), days from onset of recent symptoms (if any) to imaging (C: 244.6 ± 238.0; PCC: 204.3 ± 222.5) and the median number of vaccines received prior to imaging (C: 2 doses, range: 0 to 8 doses; PCC: 1 dose, range: 0 to 5 doses). There were no significant group differences in cardiovascular risk factors, which included body mass index, smoking status, and hypertension status.

**Table 1:** Demographic and clinical characteristics.

	Controls (*n* = 21)	COVID-19 (*n* = 36)	Test statistic	*p*
Demographics				
Age (years)	41.6 ± 14.7 [19, 70]	42.7 ± 10.4 [25, 59]	t = 0.33	0.74
Female	13 (62%)	26 (72%)	X^2^ = 0.35	0.95
Caucasian	17 (81%)	24 (67%)	X^2^ = 1.49	0.69
Education (years)	17.1 ± 2.8 [13, 35]	16.9 ± 2.7 [12, 23]	U = 373.5	0.94
COVID-19 Variables				
Days from the most recent onset of symptoms to imaging	244.6 ± 238.0 [31, 901] *5 participants never experienced symptoms	204.3 ± 222.5 [53, 1,291]	t = 0.59	0.56
Number of vaccines received prior to imaging [min; max]	2.3 ± 1.7 [0, 4]	1.5 ± 1.5 [0, 4]	U = 173.5	0.17
**Self-reported symptoms at time of assessment (ongoing | resolved | did not experience)**				
Fatigue	0 (0%) | 13 (62%) | 8 (38%)	17 (47%) | 17 (47%) | 2 (6%)	X^2^ = 16.51	0.01
Headache	0 (0%) | 2 (10%) | 19 (90%)	13 (36%) | 8 (22%) | 15 (42%)	X^2^ = 14.28	0.01
Fever	0 (0%) | 7 (33%) | 14 (67%)	0 (0%) | 29 (81%) | 7 (19%)	X^2^ = 15.38	0.01
Cough	0 (0%) | 11 (52%) | 10 (48%)	9 (25%) | 20 (56%) | 7 (19%)	X^2^ = 8.62	0.13
Sore throat	0 (0%) | 12 (57%) | 9 (43%)	2 (6%) | 27 (75%) | 7 (19%)	X^2^ = 4.85	0.43
Shortness of breath	0 (0%) | 6 (29%) | 15 (71%)	12 (33%) | 9 (25%) | 15 (42%)	X^2^ = 8.30	0.14
Gastrointestinal symptoms	0 (0%) | 3 (14%) | 18 (86%)	5 (14%) | 16 (44%) | 15 (42%)	X^2^ = 11.67	0.04
Changes to sense of smell and/or taste	0 (0%) | 2 (10%) | 19 (90%)	13 (36%) | 8 (22%) | 15 (42%)	X^2^ = 11.98	0.04
**Cardiovascular risk factor**				
Body mass index (BMI)	26.0 ± 6.5 [18.5, 45.2]	29.5 ± 27.9 [4.7, 187.7]	t = 0.57	0.57
Smoking (current smoker | quit smoking | never smoke)	0 (0%) | 5 (24%) | 16 (76%)	2 (6%) | 9 (25%) | 25 (69%)	X^2^ = 2.11	0.83
High blood pressure (current | resolved | did not experience)	1 (5%) | 0 (0%) | 20 (95%)	3 (8%) | 1 (3%) | 32 (89%)	X^2^ = 0.23	0.99
Diabetes (Type II) (current | resolved | did not experience)	0 (0%) | 0 (0%) | 21 (100%)	1 (3%) | 0 (0%) | 35 (97%)	X^2^ = 0.00	1.00
**NIH toolbox cognition battery (age-corrected standard scores)**				
Fluid cognition	112.3. ± 12.6 [88, 146]	99.9 ± 18.0 [72, 143]	t = 2.70	0.01
Crystallized cognition	112.3 ± 12.6 [88, 146]	99.6 ± 17.2 [72, 143]	t = 2.94	<0.01
**NIH toolbox emotion battery (T-scores)**				
Negative affect	53.8 ± 9.0 [42, 78] {1}	58.8 ± 9.0 [42, 78] {3}	t = 1.96	0.06
Social satisfaction	45.8 ± 7.6 [29, 56] {1}	46.1 ± 10.0 [23, 66] {3}	t = 0.13	0.90
Well-being	46.1 ± 10.0 [23, 66] {1}	46.1 ± 10.0 [23, 66] {3}	U = 250.5	0.15

Data are presented as mean ± standard deviation, [min, max], or count (number or %). Between-group comparisons were performed using independent samples t-tests or Mann–Whitney U-tests for continuous data, and chi-squared tests or Fisher’s exact tests for categorical data (i.e., binary categories; and ongoing vs. resolved vs. did not experience). Significant differences were set at an uncorrected *p* < 0.05. Numbers in braces indicate participants with missing/faulty data.

There were significant group differences in five of eight self-reported symptoms at the time of assessment (Table 1 shows data as current / resolved / did not experience): fatigue, headache, fever, gastrointestinal, and changes to smell and/or taste (*p* > 0.05). Cough, sore throat, and shortness of breath were not different between groups. There were significant differences in NIH scores for fluid cognition and crystallized cognition (*p* < 0.01), whereas the three emotion assessments showed no group differences (*p* > 0.06).

### Inspection of the DSC AIF

**Figure 1:**
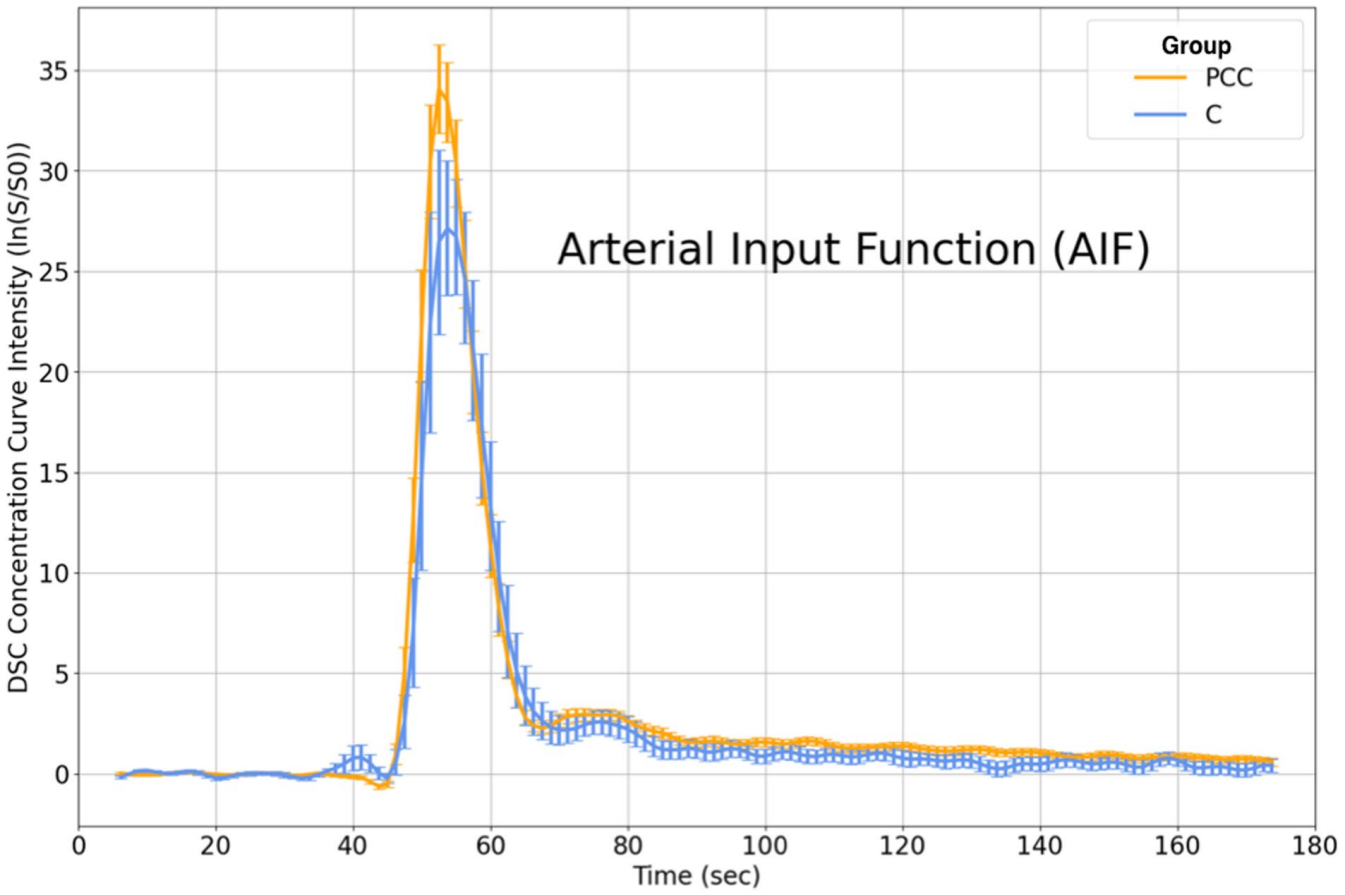
DSC concentration curves show the average response within PCC and C groups for the AIF time series. Visually and relative to the C group, the PCC group showed an increased peak and slightly elevated concentration curve beyond the first passage of the bolus. DSC = Dynamic Susceptibility Contrast; PCC = post COVID-19 condition; C, control; AIF, arterial input function.

The AIF calculation produced characteristic time series for PCC and C groups (Figure 1). Each curve represents the group average, with error bars indicating the standard error after winsorizing at the 5th and 95th percentiles. AIF curves exhibited a typical peaked appearance during the first passage of the DSC bolus (~ 45-65 s). The PCC group displayed slightly higher AIF values than the C group, from bolus onset to the peak concentration. This trend of elevated AIF values in the PCC group persisted, albeit to a lesser degree, after bolus passage.

### Results from group differences in DSC rCBV and rCBF

**Figure 2:**
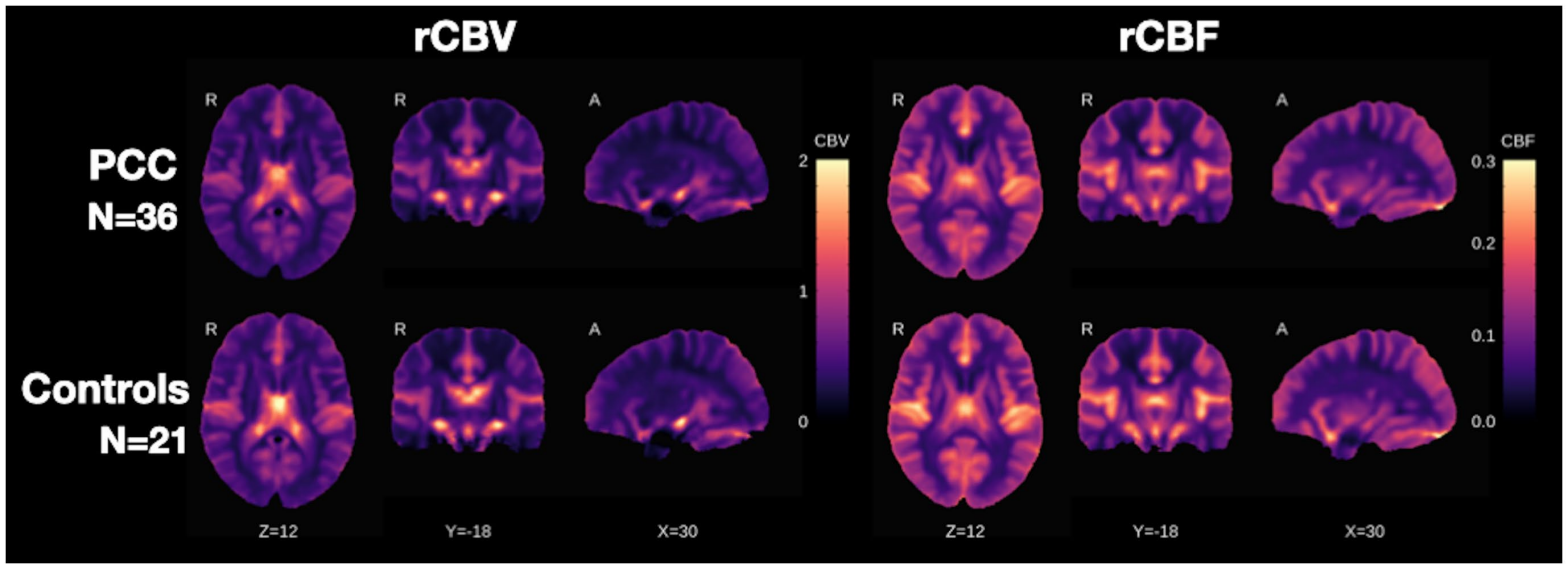
Average rCBV (left) and rCBF (right) maps are shown in three orthogonal views for the two groups under study (PCC and C). The color bar denotes the rCBV or rCBF normalized units relative to primary visual cortex reference. The cross-sectional views are defined by *x*, *y*, and *z* coordinates in the MNI standard space. rCBV, relative cerebral blood volume; rCBF, relative cerebral blood flow; PCC, post COVID-19 condition; C, control; MNI, Montreal Neurological Institute.

Average rCBV and rCBF maps were generated for both groups and are shown in three orthogonal views (Figure 2). Visual inspection reveals a consistent contrast between grey and white matter across groups.

**Figure 3:**
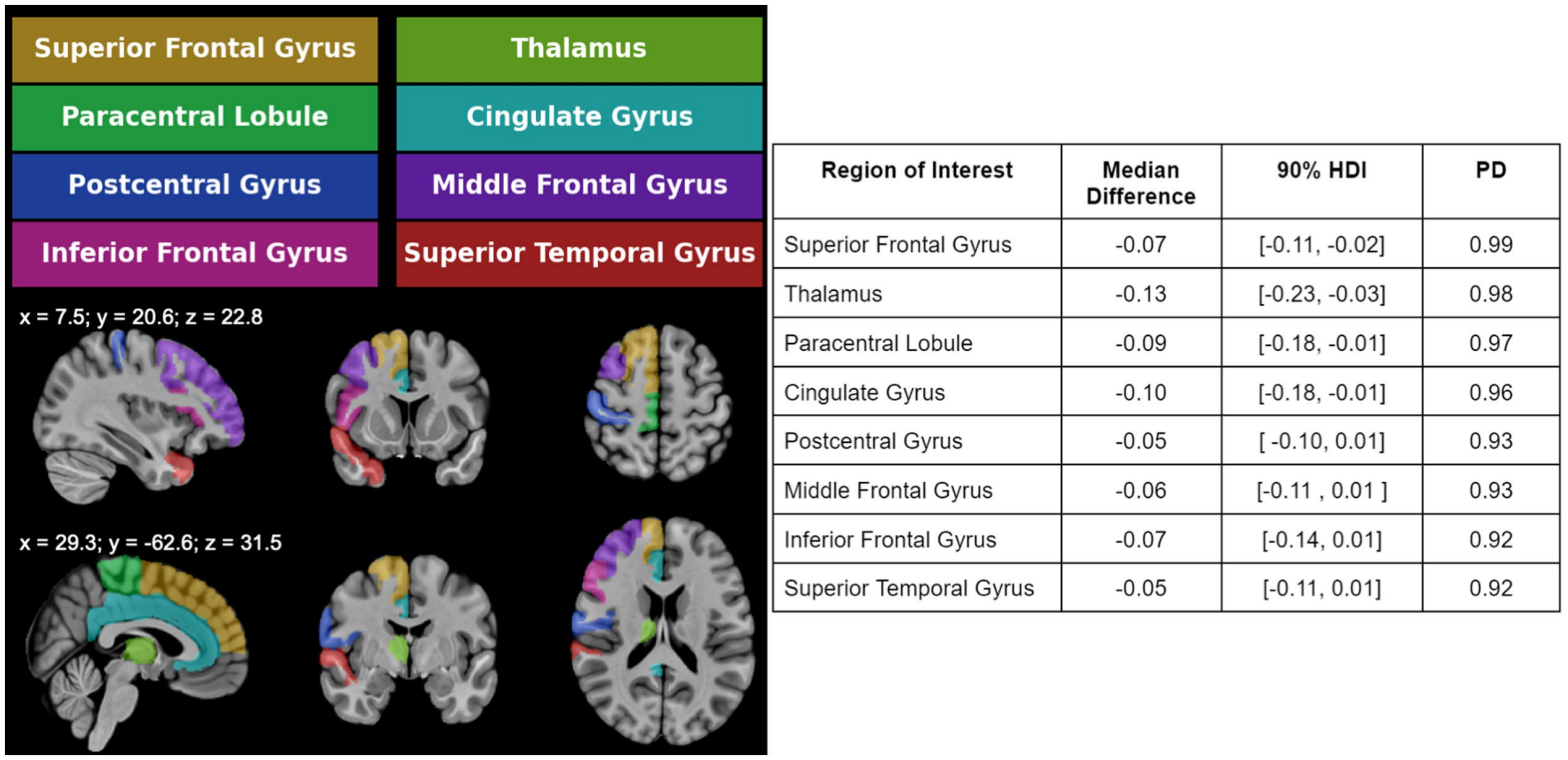
Left: Atlas-based ROIs are shown on one brain hemisphere for visualization purposes. The ROIs in color showed significant rCBV group differences. Right: The ROIs are listed alongside the median between-group difference, 90% HDI, and PD values. Eight ROIs showed rCBV decrease in PCC compared to C, as reflected by the negative value for the median group difference. No ROI showed an rCBV increase for PCC compared to C participants. ROI = region of interest; rCBV, relative cerebral blood volume; HDI, highest density interval; PD, probability of direction; PCC, post COVID-19 condition; C, control.

The median group differences and accompanying 90% HDI values were estimated from each of the eight ROIs exhibiting reduced rCBV in the PCC group, with the PD values to indicate the direction of the group difference (Figure 3). The rCBV values were consistently lower in PCC compared to controls. Affected regions included the superior frontal gyrus, thalamus, paracentral lobule, cingulate gyrus, postcentral gyrus, middle frontal gyrus, inferior frontal gyrus, and superior temporal gyrus. These decreases were predominantly located in anterior frontal and cingulate areas, with some involvement of temporal and subcortical ROIs. Additional details on the group-level differences are reported in the supplementary table.

In terms of the regional rCBF values in the secondary analysis, there were no ROIs that met the predefined criteria for a high PD, hence no rCBF group differences. As reflected in the supplementary table, there were trends toward lower rCBF in PCC compared to controls, albeit the group differences had low statistical certainty.

## Discussion

The present study examined whether PCC exhibit neurophysiological changes in rCBV and rCBF compared to controls. Regional decreases in rCBV were revealed in the PCC group but there were no group differences in rCBF. Groups were similar in demographics, vascular risk factors, days from symptom onset to imaging, and vaccination history. However, the PCC group reported a higher prevalence of fatigue, headache, fever, gastrointestinal symptoms, and altered smell/taste, and performed worse on measures of fluid and crystallized cognition.

Eight ROIs exhibited reduced rCBV in PCC, which reflects impaired perfusion, and aligns with prior CBF findings using ASL (Kim et al., 2023). Decreased rCBV was observed in the superior, middle, inferior frontal gyri, paracentral lobule, cingulate gyrus, and postcentral gyrus. The superior frontal gyrus has shown reduced CBF between PCC and control groups based on ASL (Ajčević et al., 2023). The middle frontal gyrus showed lower ASL CBF in non-hospitalized COVID-19 participants from one study (Sen et al., 2023), and altered resting-state ASL-based functional connectivity in another study involving patients 6 months recovered after COVID-19 hospitalization (Li et al., 2023). The inferior frontal gyrus exhibited reduced grey matter volume two years after COVID-19 infection in comparison to controls (Du et al., 2023) and suggests that neurophysiological changes may contribute to longer-term structural changes. The paracentral lobule and cingulate gyrus are part of a regional brain network implicated in fatigue (Wylie et al., 2020), which is a common PCC symptom (Lopez-Leon et al., 2021).

Reduced rCBV in the superior temporal gyrus aligns with prior evidence of gray matter reductions in this region among non-hospitalized COVID-19 individuals relative to non-infected controls (Douaud et al., 2022), and as reflected by converging neuroimaging evidence in a recent meta-analysis (Guo et al., 2024). The thalamus was the only subcortical region with decreased rCBV in PCC, consistent with findings of altered neuroanatomical texture and connectivity in PCC (Churchill et al., 2024; Churchill et al., 2023). The thalamus is implicated in fatigue in multiple sclerosis (MS) (Capone et al., 2020) and may play a role in PCC. Lower thalamic rCBV in MS has been linked to elevated neurofilament light chain, a marker of brain injury (Jakimovski et al., 2020).

The observed rCBV reductions may reflect underlying pathophysiological mechanisms, including neurovascular uncoupling, dysautonomia, altered oxygen extraction, blood–brain barrier disruption, and neuroinflammation (Zhao et al., 2023; Greene et al., 2024; Braga et al., 2023). To provide context for the latter, positron emission tomography (PET) and resting state functional MRI studies have revealed that gliosis and functional activation are examples of neuroimaging readouts that relate to inflammation markers; and these previous findings were relevant to cognitive and mood symptoms (Braga et al., 2023; Guo et al., 2024). Early and late-phase blood markers, such as neurofilament light chain, have also indexed brain injury that correlated with neurocognitive symptoms (Michael et al., 2023; Gutman et al., 2024).

PCC individuals in the present study were not hospitalized during the acute phase of COVID-19, emphasizing that PCC-related alterations to brain physiology may occur irrespective of the acute presentation of illness. Conversely, in people hospitalized due to COVID-19, ASL-based perfusion abnormalities were reported in 17 of 26 patients (Helms et al., 2020) and in all 11 participants of a separate MRI study (Helms et al., 2020), along with microvascular abnormalities in grey matter (van der Knaap et al., 2025). Among outpatients that were recruited from a post COVID-19 neurological clinic and roughly one decade older than the current PCC sample, there were widespread ASL-based perfusion decreases in the COVID-19 group compared to controls (Ajčević et al., 2023). It is noteworthy that two studies in this set reported hypoperfusion in frontal and temporal regions (Helms et al., 2020; Ajčević et al., 2023), aligning to some extent with the ROI findings from the present study.

The DSC-rCBF data showed reduced levels in PCC compared to controls, for some ROIs, but did not reach the cutoff for probability of direction. The rCBF null group findings are contrary to prior PCC studies using ASL-CBF (Kim et al., 2023; Ajčević et al., 2023). In terms of the biophysics, moderate to high DSC-rCBF and ASL-CBF agreement is expected given a systematic review; however, the literature comparing these techniques is based largely from stroke and large artery disease cohorts (Liu et al., 2021). One advantage of DSC is the high signal-to-noise ratio from injected contrast agent, yet it is recognized that DSC-derived rCBF is sensitive to the AIF shape and partial volume effects (Zaharchuk et al., 2010). The rCBV maps are considered more robust than the rCBF maps because the former is calculated as the area under the tissue concentration curve divided by the area under the AIF curve. The DSC-rCBF mapping instead relies on block-circulant deconvolution that can be sensitive to noise. Future work comparing DSC-derived rCBF and ASL CBF maps in PCC is warranted, which could evaluate analysis methodologies, advanced AIF modeling, and pre-bolus injection to improve rCBF quantification. Regarding regional cerebrovascular adaptations, the current findings suggest regional vasoconstriction in PCC, resulting in lower rCBV. A more muted group difference in rCBF, despite lower rCBV, would be an indication of reduced regional mean transit time.

The present study highlights that there are opportunities to further characterize the neurovascular underpinnings associated with PCC. It would be interesting to evaluate rCBV, rCBF, and other brain imaging measures available from the study protocol in relation to the clinical, cognitive, and emotions data; however, this is beyond the scope of the current work. One prior study has revealed that perfusion to medial frontal and subcortical brain regions was altered in relation to olfactory dysfunction in a PCC sample (Yus et al., 2022). Given a large enough sample, partial least squares or canonical correlation analysis could be used as statistical approaches to study brain-to-behaviour relationships (Shirzadi et al., 2023). DSC-MRI is used clinically to assess brain conditions such as stroke and tumors in individual patients. The present work shows DSC findings that were different between PCC and controls at a group level. Ultimately, insight from the research findings may lead to knowledge exchange between researchers, clinicians, and individuals impacted by PCC.

This study has some limitations. This study was limited by the relatively small number of participants that underwent DSC MRI. This technique requires the administration of an exogenous gadolinium-based contrast agent, which is not indicated for individuals with impaired kidney function or an allergy to the contrast agent. The sample size was similar to other functional and/or neurophysiological imaging studies (Braga et al., 2023; Klironomos et al., 2020), but considerably smaller than some other neuroimaging COVID-19 studies (Douaud et al., 2022), hence some caution is warranted in interpreting the current findings. Second, correlations of rCBV with clinical assessments (cognitive, affective and symptom-related) and blood biomarkers (e.g., neurofilament light) would be of interest, but beyond the current scope. Third, the PCC group had a slightly higher AIF peak compared to controls, which could have influenced rCBV and rCBF values. It would be interesting to better understand the intraluminal factors that could have influenced the arterial hemodynamics and/or AIF, as noted with acute perfusion imaging in COVID-19 (Mohamud et al., 2020). Prior work shows that DSC-derived maps are relatively consistent across different DSC software packages, therefore the current findings are not expected to be influenced by the chosen analysis (Kudo et al., 2013). However, it is not possible to rule out potential operator bias in the AIF estimate, and future work could include automated approaches, such as clustering-based, component analysis, or other data-driven heuristics. Fourth, some controls were infected with COVID-19 whereas others were infected by another flu-like illness. Heterogeneity in the controls arising from COVID-19 infection is unlikely to be a significant confounding factor, however, based on a large study (Hampshire et al., 2024). Lastly, data were unavailable to report heterogeneity in the coronavirus strain across the PCC group (e.g., alpha, delta, omicron), precluding analysis on this matter.

## Conclusion

The current study adds to the growing body of COVID-19 and brain imaging literature by revealing group-level rCBV differences, capitalizing on DSC-MRI in a substantial sample of individuals with PCC or well-matched controls (Zhao et al., 2023). Participants from both groups were assessed with DSC-MRI several months after self-isolation for acute COVID-19 infection or a non-COVID-19 illness. The findings revealed a pattern of lower rCBV in the PCC group relative to controls in ROIs that have previously been reported in analogous structural and perfusion neuroimaging studies. The group-level differences in rCBV reached a high degree of statistical certainty, whereas the rCBF showed a similar trend but lower statistical certainty. These findings help to reveal that neurophysiological changes are associated with PCC symptoms, and that further research is needed in this area.

## Data Availability

The raw data supporting the conclusions of this article will be made available by the authors, without undue reservation.
