# Supplementary material for: Post COVID-19 condition is associated with altered regional cerebral blood volume as revealed by dynamic susceptibility contrast MRI
Source: Front Neuroimaging. 2026 Feb 18;4:1688973. doi: 10.3389/fnimg.2025.1688973 (PMC12956688; doi:10.3389/fnimg.2025.1688973)
Supplement: Supplementary file 1 [file Data_Sheet_1.PDF]

Date of Visit: __ __/__ __/__ __ __ __ [DD/MM/YYYY]

Date of testing and results (DD/MM/YYYY)	1. ______________ Positive ☐ Negative ☐<br>2. ______________ Positive ☐ Negative ☐<br>3. ______________ Positive ☐ Negative ☐<br>4. ______________ Positive ☐ Negative ☐	
Has documentation of test results	No ☐ Yes* ☐ specify: ___________<br>*Attach de-identified copy of documentation	
Date of onset of symptoms	(DD/MM/YYYY)	
Description of symptoms	Fever ☐1	ongoing ☐1 Resolved ☐2 **no change** ☐3
	cough ☐2	ongoing ☐1 Resolved ☐2 **no change** ☐3
	sore throat ☐3	ongoing ☐1 Resolved ☐2 **no change** ☐3
	shortness of breath ☐4	ongoing ☐1 Resolved ☐2 **no change** ☐3
	Excessive fatigue ☐5	ongoing ☐1 Resolved ☐2 **no change** ☐3
	GI symptoms ☐6	ongoing ☐1 Resolved ☐2 **no change** ☐3
	changes to sense of smell and/or taste ☐7	ongoing ☐1 Resolved ☐2 **no change** ☐3
	Other:<br>_______________________________<br>_______________________________	ongoing ☐1 Resolved ☐2 **no change** ☐3<br>ongoing ☐1 Resolved ☐2 **no change** ☐3
Hospitalization	N/A ☐ Yes ☐ Location:_____________________<br>Admission date: _______________<br>Discharge date: _______________	
Other Investigations* (add date and location if known)	Blood work No ☐ Yes ☐ specify: _____________________<br>Chest x ray No ☐ Yes ☐ specify: _____________________	

Date of Visit: __ __/__ __/__ __ __ __ [DD/MM/YYYY]

	Saturation levels No ☐ Yes ☐ specify: ____________________<br>CT scan No ☐ Yes ☐ specify: ____________________<br>Other No ☐ Yes ☐ specify: ____________________<br><br>*Sign release of information to obtain access to medical records

Travelled outside of Canada in the last 14 days	☐ Yes ☐ No
Had close contact with a confirmed or probable case of COVID-19	☐ Yes ☐ No
Had close contact with a person with acute respiratory illness who travelled outside of Canada in the last 14 days	☐ Yes ☐ No
Have been maintaining social distance.	☐ Yes ☐ No

CRF completed by:

Name: ________________________________ Signature: _________________________________
